# Written Language Production in Children With Developmental Language Disorder

**DOI:** 10.3389/fpsyg.2022.833429

**Published:** 2022-03-10

**Authors:** Georgia Andreou, Vasiliki Aslanoglou

**Affiliations:** Department of Special Education, University of Thessaly, Volos, Greece

**Keywords:** written language difficulties, text, developmental language disorder, Greek language, writing performance

## Abstract

This study contributes to the cross-linguistic investigation of written language difficulties in children with DLD by reporting new findings from Greek-speaking individuals. Specifically, we investigate the writing performance of children with DLD and compare it to that of a group of typically developing (TD) children, matched for gender and chronological age. The specific orthographic properties of Greek, radically different from those of English, offer a unique opportunity to understand the nature of written language production in DLD. The participants of the study were 62 children, 31 with DLD and 31 TD. Both groups were asked to write a text on a special prompt they were given by the researcher and they were assessed in the total number of words used in text, in the proportion of incorrectly spelt words in text as well as in the use of verbs, nouns, content and function words. Also, the different words and the total number of main and subordinate clauses each of the participants used in their text were counted. The findings of the study showed that the written outputs of the DLD group were poorer in almost all measurements compared to those of their TD peers. We discuss our findings in relation to those reported by other languages, in particular English, and spell out the implications for assessing written language in children with DLD.

## Introduction

Previous research has proven that about 7.4% of the population has significant language deficits (8% boys and 6% girls; Tomblin et al., 1997; Norbury et al., 2016) which cannot be attributed to any obvious etiology, such as hearing loss, low non-verbal intelligence, or any neurological disorder (Haebig et al., 2016; Montgomery et al., 2016). The population presenting those characteristics is classified as having Developmental Language Disorder (DLD).

Developmental language disorder is a neurodevelopmental disorder of language (Sengottuvel and Rao, 2015) and in almost all cases the diagnosis is made at pre-school age. The term DLD, previously known as Specific Language Impairment (SLI), is recent and has been developed by the CATALISE team (Bishop et al., 2016). The experts of the team argued that environmental and biological factors, such as family history, socio-economic status, or problems while birth, should not be considered as exclusion criteria for DLD. In addition, DLD may co-exist with other neurodevelopmental disorders and therefore an individual should not be banned from inclusion in a DLD group when there is a diagnosis of attention-deficit, hyperactivity, speech and behavioral problems, or low non-language ability. Also, according to the CATALISE group, DLD does not necessarily require any differentiation between verbal and non-verbal intelligence.

According to researchers, children with DLD do not present a consistent language profile since their language characteristics show great heterogeneity (Pijnacker et al., 2017). Previous studies have documented difficulties on the part of the DLD population in non-word repetition (Bishop et al., 1996; Baddeley et al., 1998; Larkin et al., 2013; Lalioti et al., 2016; Mengisidou et al., 2020), in verbal short-term memory (Hill, 2001; Girbau and Schwartz, 2007) as well as impairments in terms of working memory and processing speed (Archibald and Gathercole, 2006; Leonard, 2014). In addition, they face problems in phonological awareness and in oral language production, including many grammatical errors. Children with DLD also experience attention and reading difficulties, they find it difficult to understand both complex and simple sentence structures (Montgomery et al., 2016) and they have difficulties in understanding texts while reading (Talli et al., 2016). Moreover, children with DLD produce fewer verbs while writing as compared to their typically developing (TD) peers (Stuart et al., 2020). The deficits they face are at a morphological, semantic, and pragmatic level while the phonological processes this population uses are similar to those of typically developing younger children (Leonard, 2014).

Research on DLD in the past years has focused mainly on oral language since individuals with DLD present serious problems in oral language production. Fewer researchers worked on characteristics of the written language in DLD. Previous research on the written language production of individuals with DLD has reported difficulties in their writing performance, which is characterized by poorer texts concerning the amount of words in total or in the total number of different words used, spelling errors as well as omissions of whole words or omissions of grammatical morphology (Gillam and Johnston, 1992; Windsor et al., 2000; Mackie and Dockrell, 2004). It is very likely that difficulties in writing reflect the difficulties shown in the oral language of individuals with DLD.

A number of studies concerning written language production, most of which conducted in the English language, have focused on omissions of grammatical morphology and especially on the production of regular plural number marker -*s* and regular past tense marker -*ed* in children’s writing (Windsor et al., 2000; Mackie and Dockrell, 2004; Larkin et al., 2013) concluding that children with DLD tended even to omit both the regular plural marker and the regular past tense marker as compared to their typically developing (TD) peers of a corresponding chronological age or to their peers of a corresponding linguistic age. Also, children with DLD tended to omit whole words mostly related to the auxiliary verb *be* while writing as compared to their peers of a corresponding chronological or linguistic age (Windsor et al., 2000; Mackie and Dockrell, 2004). Additionally, in their scoping review, Broc et al. (2021) focused on the nature of spelling errors in DLD children. The researchers separated the studies included in their paper in order to draw their conclusions. More specifically they divided them into studies containing dictation tasks and studies containing written narratives while simultaneously, they took into consideration the specific language each study carried out highlighting the type of orthography as an important factor (opaque or transparent orthographic system). Concerning the dictation and narrative tasks, children with DLD produced more phonologically unacceptable spelling errors. These errors varied by age and by the nature of the words dictated. The DLD group in high school produced fewer and less phonologically unacceptable spelling errors as compared to their TD peers of a corresponding chronological age, but they tended to produce phonologically unacceptable errors in higher proportion for an extended period of time. Moreover, Werfel and Krimm (2015) studied the number and the type of spelling errors children with DLD tented to produce in their written language production as compared to a group of TD children of a corresponding chronological age. According to the results of the study children with DLD used a significantly smaller number of correct words while writing as compared to those of their TD peers of a corresponding chronological age. In addition, children with DLD used to omit letters or made incorrect use of letters in written language production.

Several researchers studied the total production of words of children with DLD while writing, using different control groups in order to identify if there was a significant difference among them or not. Mackie and Dockrell (2004) and Stuart et al. (2020) agreed *via* their studies that DLD group produced fewer words in their written texts than TD children of a corresponding chronological age but not than those of a corresponding linguistic age. Dockrell et al. (2007) concluded the same in their study in which only children with DLD participated and measurements were repeated twice within a gap of 2 years. Those measurements revealed that DLD children produced short texts in both times of testing. Ralli et al. (2021) in their research in Greek indicated that children with DLD performed worse than their TD peers in measures concerning productivity and especially, in the total number of written words produced.

In addition, lexical diversity was measured in several studies (Machie et al., 2013; Williams et al., 2013; Stuart et al., 2020) revealing that the DLD group used a limited number of different words in their written texts. Williams et al. (2013) found that the DLD group used a significantly less diverse range of words than their TD peers of a corresponding chronological age. The group of spelling age-matched children were not significantly different from the other two groups that participated in the study. Furthermore, Machie et al. (2013) designed a study that included four groups of children. Specifically, a DLD group, a chronologically age-matched group, a group of children who was matched with the DLD group according to the results of a receptive vocabulary test and a group of children who was matched according to the results of reading decoding tests. The results of their study revealed that children with DLD performed worse in comparison to chronologically age-matched children in all measures of writing (number of words overall, words per minute, different words used, word omissions, and misspellings). However, their performance was comparable to that of the group matched in terms of receptive vocabulary on measures of sentence complexity and productivity. Also, Ralli et al. (2021) in their study in Greek concluded that the DLD group differed significantly from their TD peers of a corresponding chronological age in the number of different words used.

Moreover, several studies in DLD focused on the relation between the oral language and spelling. Windsor et al. (2000) asked their participants to narrate orally two stories and, also, to write two more stories based on a video. The results of the study revealed that children with DLD made more errors in their written language production than their oral language production as compared to their TD peers of the same chronological and linguistic age. Those errors concerned mainly verb rather than noun types. Furthermore, Gillam and Johnston (1992) explored the relationship between oral and written language production of children with DLD using four experimental groups. The results of the study showed that the DLD group and the reading-matched group used more complex linguistic forms in their orally produced stories than in their written ones. In contrast, the age-matched and the language-matched groups used more complex linguistic forms in their written stories.

Machie et al. (2013) carried out a study in order to examine the written language production of children with DLD. The DLD group produced less complete sentences compared to the three control groups (the first one consisted of a corresponding chronological age, the second one consisted of children who were matched according to receptive vocabulary and the third one consisted of children who were matched according to reading decoding). In addition, Williams et al. (2013) pointed out that the organization, unity, and coherence of the texts composed by children with DLD were poorer compared to those of their TD peers of a corresponding chronological age and, also, to their peers of a corresponding linguistic age. Moreover, Favart et al. (2016) studied the ability of children and adolescents with DLD to manage cohesion while writing a narrative in a communicative situation in French as compared to their TD peers of a corresponding chronological age and concluded that the texts of the participants with DLD were shorter than those of their TD peers in both age groups. The majority of children with DLD (60%) did not use any connectives in contrast to their TD peers who used connectives at a satisfactory level. Also, children with DLD never used more than two categories of connectives and they usually used the “*and*” connective and chronological connectives.

Research in DLD, also, focused on the reading performance of children with DLD which is supposed to be a predictor of their performance in written language production (Dockrell et al., 2007), while reading difficulties of children with DLD have a significant impact on their spelling scores (Joye et al., 2019). Mackie and Dockrell (2004) concluded that children with DLD produced a significantly higher number of syntactic errors as compared to their peers of a corresponding chronological age as well as to the group of a corresponding language age. It was observed that the higher the score they obtained on the word reading task, the lower the proportion of spelling errors in their writing task was. Additionally, Larkin et al. (2013) in their research with a group of 15 DLD children, a group of 15 TD children matched for age, and a group of 15 spelling age-matched children pointed out that single word reading has a particularly powerful association with DLD children’s written language ability.

All the studies mentioned above concerned research conducted mainly in the English language. According to Leonard (2014), the difficulties faced by children with DLD are associated with the characteristics of their language. Greek is a language which is transparent in terms of reading and opaque in terms of writing (Mouzaki and Protopapas, 2010). That means that students often find difficulties in writing as the Greek spelling system is characterized by a high degree of complexity. For example, the phoneme /i/ corresponds to more than one graphemes depending on the circumstances (/ι/, /η/, /ει/, /οι/, /υ/; Karatzas, 2005). Also, Aidinis (2012) pointed out that a great asymmetry in Greek between writing and reading is observed because words are read correctly based on the way they are written, but their writing may not be correct if we rely only on the way they are pronounced (Mouzaki and Protopapas, 2010). In addition, the Greek language is characterized by rich morphology and the morphemes are utilized in order to create words, to show the gender, the case and the number of a noun or the person, and the number (singular or plural) of a verb (Andreou, 2012).

The only study that was conducted in the Greek language concerning written text production in DLD children was that of Ralli et al. (2021). In this study, written text production (productivity, accuracy, and complexity) was examined in relation to oral language, cognitive, visual-motor coordination, and handwriting skills in Greek-speaking children with DLD (*N* = 30) as compared to a group of 30 TD children of a corresponding chronological age. The participants attended the second grade of primary school and they were asked to write a story based on a special prompt within 5 min. Their texts were evaluated in terms of productivity, accuracy, and complexity. *Productivity* was measured through the calculation of the number of the written words used and the number of different words produced. A*ccuracy* was measured through the (a) percentage of spelling errors among the total number of words written, (b) percentage of lowercase capital letters errors among the total number of words written, (c) percentage of stress mark errors among the total number of words written, and (d) percentage of subject–verb agreement errors among the total number of subject–verb pairs produced. C*omplexity* was measured through the (a) number of main and subordinate clauses produced in text, (b) percentage of subordinate clauses among the total number of clauses produced, (c) number of coordinating clauses. Children with DLD performed worse than their TD peers in measures concerning productivity and especially, in the total number of written words and in the total number of different words produced. In terms of writing accuracy, the DLD group and the TD group did not differ significantly in the percentage of lowercase–capital letters errors. In addition, concerning writing complexity the two groups did not differ significantly in the number of coordinating clauses and the percentage of subordinate clauses produced.

Since only one study has been conducted concerning written language production among the DLD population in Greek, further research in the Greek language is needed in order to find out whether the difficulties the DLD population faces in other languages, different in terms of reading and writing, are also encountered in Greek.

## Materials and Methods

### Participants

A total of 62 children took part in our study. The sample consisted of 31 children, 24 males, and 7 females, who were diagnosed with DLD (mean age 7;6 years old, SD: 7.85, range: 81–119 months) and 31 typically developing children (TD) who served as controls (mean age 7;7 years old, SD: 6.63, range: 82–105 months) matched for age and gender with the DLD group. Typically developing children were recruited from Greek state schools and selected by their teachers on the basis of curriculum assessments and also as having no additional learning difficulties. Children with DLD were recruited from speech therapists’ offices. Children of both groups attended the 2^nd^ and 3^rd^ grade of primary school.

The parents of the DLD group were asked by the researcher to fill in a questionnaire, in cooperation with the speech therapist, including questions about demographic characteristics of their family (job, education etc.) as well as characteristics of their children, including difficulties facing at school and characteristics of their language development (for example, if they face any difficulties while communicating with others orally, if their vocabulary is limited). These questionnaires were completed and were given afterwards to the examiner in order to record the particular answers. The results of the questionnaire, as shown in Table 1, revealed the following: DLD children were monolinguals (Greek Language) and their parents (mother and father) were of Greek nationality, speaking only the Greek language at home. The DLD participants did not present any hearing loss or visual impairment or any known neurological disorder and were all at about the same socio-economic status. At least one of DLD parents had a job and had graduated from at least elementary school. The DLD children were diagnosed by special therapists working only in public and not in private centers. All children attended public schools and had an official diagnosis describing in detail their language problems/deficits. Also, all participants of the DLD group participated in speech and language therapy at private centers and worked individually with the speech and language therapist and not in groups. The entire DLD group was reported to show persistent language disorders at the time of testing and that was the reason they attended private centers for speech and language therapy. In addition, the DLD group was reported to face problems with the language lesson at school and difficulties especially in reading or writing. They did not attend special schools or classes in their public schools and no member of their family had special educational needs. The DLD participants showed normal non-verbal IQ abilities according to the measurements reported in their files.

**Table 1:** Demographic characteristics of the DLD and TD group.

Reported Background Information	DLD Group (*n* = 31, CA = 7;6 Males = 24, Females = 7)	TD Group (*n* = 31, CA = 7;7 Males = 24, Females = 7)
Attending public schools	√	√
Having an official diagnosis for language problems/deficits	√	X
Attending private centers for speech and language therapy	√	X
Showing language disorder in the past years	√	X
Showing language disorder at the time of testing	√	X
Monolingual students	√	√
Parents (mother and father) of Greek nationality	√	√
Problems reported with the language lesson at school	√	X
Having hearing loss or visual impairment	X	X
At least one parent having a job	√	√
Parents graduated at least from elementary school	√	√
Attending special schools or classes in public schools	X	X
Having other family members with special educational needs	X	X
Facing any known neurological disorder	X	X
Facing problems in reading or writing	√	X
Normal non-verbal IQ abilities	√	√

*√ = Yes, X = No; DLD, Developmental Language Disorder, TD, Typical Development*.

### Materials and Procedure

**Figure 1:**
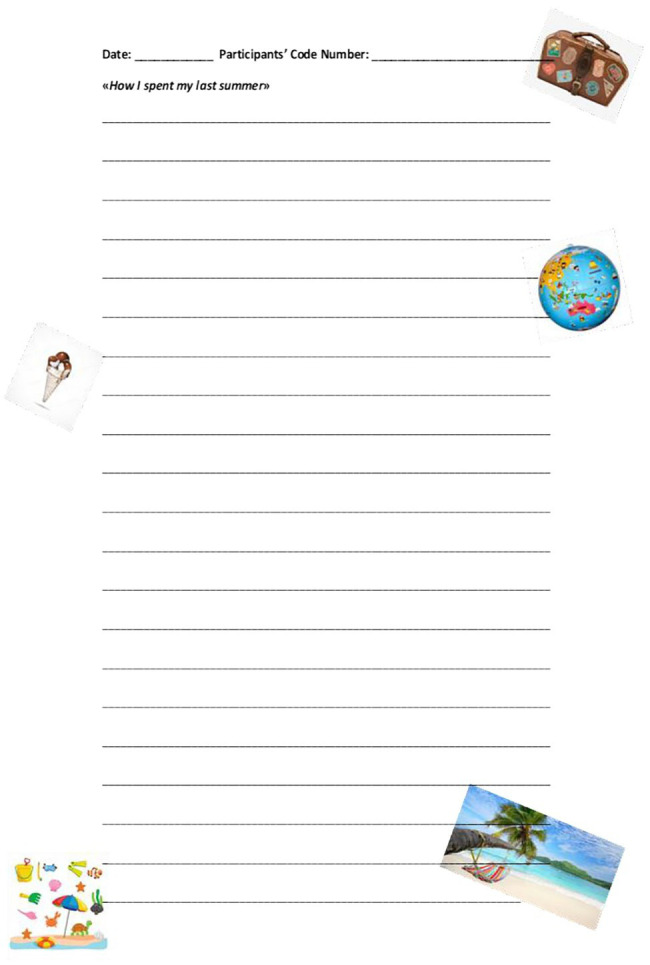
The answer sheet given to each participant individually.

The participants of both groups were asked to write a text on the following prompt “How I spent my last summer” within 15 min (Figure 1). This prompt elicits narrative style writing and is appropriate for our participants regarding to their chronological age and the curriculum. The variables measured in the writing task were the (a) total number of words, (b) percentage of incorrectly spelt words among the total number of words in text, (c) total number of different words, (d) total number of nouns or verbs used, (e) total number of clauses (main and subordinate) and percentage of subordinate clauses among the total number of clauses produced, (f) total number of content and function words.

For the purposes of the study, the children with DLD were assessed individually in a quiet room of their speech therapists’ offices while children of typical development were tested in small groups in a quiet classroom of their school. Both groups had the written consent of their parents. In particular, the researcher gave each participant an individual answer sheet. In case the participants did not have time to complete the writing, no extra time was given. During the test, the researcher did not give further explanations to the participants and did not answer any questions about whether their writings were correct or not. The total time (15 min) was sufficient and none of the participants asked for more time. The scripts of the participants were coded and were assessed by the researcher who used a separate coded log sheet for each participant in order to write down the exact numbers of variables measured.

For the statistical analysis of the data, the Statistical Package for Social Sciences (SPSS) was used and more specifically the Mann–Whitney non-parametric test.

## Study Aims

Based on the above and given the paucity of research in the Greek language, the aim of the present study is to investigate written language production in a group of children with DLD in the Greek language and compare it with that of TD age-matched peers.

The hypotheses of the study are that the performance of DLD group will be weaker than that of TD group in their written texts with respect to the following measures: (a) Total number of words, (b) Percentage of incorrectly spelt words among the total number of words in text, (c) Total number of different words, (d) Total number of nouns or verbs used, (e) Total number of clauses (main and subordinate) and percentage of subordinate clauses among the total number of clauses produced, (f) Total number of content and function words.

## Results

The results of the study, as presented in Table 2, showed that there was a statistically significant difference in the total number of words the two groups wrote (DLD M = 38.71, SD = 35.06; TD M = 46.32, SD: 28.18; *p* = 0.04) as well as in the percentage of incorrectly spelt words among the total number of words in text (DLD M = 42.77, SD = 20.74; TD M = 21.07, SD: 13.83; *p* = 0.001). Also, there was a statistically significant difference in the total number of different words in texts between the DLD and TD group (DLD M = 25.97, SD = 20.16; TD M = 34.48, SD: 16.35; *p* = 0.01). In addition, a statistically significant difference in the total number of nouns in text was found (DLD M = 9.35, SD = 8.07; TD M = 12.45, SD: 7; *p* = 0.02) but not in the total number of the verbs they used in their written texts (DLD M = 7.94, SD = 6.47; TD M = 8.77, SD: 5.08; *p* = 0.23). There was no statistically significant difference in measures that were related to the use of the total number of clauses (main and subordinate) in the texts of both groups (DLD M = 7.87, SD = 6.47; TD M = 8.74, SD: 5.12, *p* = 0.23) or in the percentage of the subordinate clauses among the total number of clauses produced (DLD M = 11.54, SD = 18.73; TD M = 13.05, SD: 18.45; *p* = 0.57). Finally, there was a statistically significant difference in the total number of content words (DLD M = 21.70, SD = 19.15; TD M = 27, SD: 15.35; *p* = 0.02) but not in measures that were related to the use of function words (DLD M = 17.39, SD = 16.74; TD M = 19.32, SD: 13.35, *p* = 0.18).

**Table 2:** Descriptive statistics for the written text production measures per group.

Variables	DLD (*n* = 31)			TD (*n* = 31)			
	M	SD	Range (min. & max. Scores represent raw scores)	M	SD	Range (min. & max. Scores represent raw scores)	*U*-test
Total number of words in text	38.71	35.06	8–162	46.32	28.18	9–117	332^*^
Incorrectly spelt words among the total number of words in text (%)	42.77	20.74	3–109	21.07	13.83	1–20	170^***^
Total number of different words in text	25.97	20.16	7–99	34.48	16.35	9–74	292^**^
Total number of nouns in text	9.35	8.07	2–36	12.45	7	2–29	308.5^**^
Total number of verbs in text	7.94	6.47	2–31	8.77	5.08	2–22	395
Total number of clauses	7.87	6.47	2–31	8.74	5.12	2–22	396.5
Subordinate clauses %	11.54	18.73	0–12	13.05	18.45	0–6	443
Total number of content words in text	21.70	19.15	5–90	27	15.35	7–68	305^*^
Total number of function words in text	17.39	16.74	3–72	19.32	13.35	1–52	384.5

DLD, Developmental Language Disorder, TD, Typical Development; *p < 0.05, **p < 0.01; ***p < 0.001.

## Discussion

The purpose of this study was to investigate written language production among children with DLD and their typically developing age-matched peers. Our aim was to compare the written performance between the two groups and it was expected that the written texts of the DLD group would be shorter, with poor word diversity and more errors compared to the TD group. The results of this study indicate that DLD students performed poorer in most of our measurements compared to their TD peers.

Our first research hypothesis was that the DLD group will present lower performance concerning the total number of words produced compared to that of their TD peers. As expected, the DLD group performed worse in this measure in comparison to TD children matched for age. This finding is in line with Dockrell et al. (2007) who studied DLD children’s written texts in terms of length, across the Wechsler Objective Language Dimensions test (WOLD; Rust, 1996) subscales and found that children with DLD produced short texts. Also, our finding is consistent with Broc et al. (2013) who measured the number of words used by their DLD and TD groups and found that the DLD group presented poorer performance than the TD peers. Our finding is also in line with the Connelly et al. (2012) study, which revealed that the DLD group produced fewer words in their writing tasks compared to their TD peers. Furthermore, our results confirm the finding of Stuart et al. (2020) in terms of the total number of words produced by the participants, while writing a text on a specific prompt and, also, are in line with Ralli et al. (2021) who carried out their study in Greek. Ralli et al. (2021) concluded that the DLD group performed worse than their TD peers of a corresponding chronological age in writing productivity measures as they wrote fewer words in their texts as compared to the TD group. It can be argued that limited performance in written language production in terms of length (total words produced) in DLD is associated with limited vocabulary knowledge (Dockrell et al., 2007). Also, writing shorter texts indicates a failure to gain access to knowledge already possessed or problems with mechanisms of writing (for example slow handwriting; Dockrell et al., 2007).

Our second research hypothesis was that the DLD group will produce a higher percentage of incorrectly spelt words among the total number of words used in text as compared to their TD peers. The results of the study revealed that the DLD group produced a higher percentage of incorrectly spelt words among the total number of words in text while writing than their TD peers. This finding is in line with previous studies (Machie et al., 2013; Williams et al., 2013; Ralli et al., 2021) which, also, found that DLD groups produced a higher proportion of spelling errors while writing than their TD peers. This finding could be attributed to the orthographic consistency which is supposed to affect the spelling accuracy (Joye et al., 2019). Especially, the Greek language is characterized by great asymmetry between writing and reading because words are read correctly based on the way they are written, but their writing may not be correct if we rely only on the way they are pronounced (Mouzaki and Protopapas, 2010). Additionally, the rich morphology of the Greek language in which the morphemes are utilized in order to create words, to show the gender, the number (singular or plural) of a verb etc. (Andreou, 2012) may cause difficulties in written language production and as a result contribute to the production of more incorrectly spelt words. The incorrectly spelt words may also reveal a fundamental weakness with linguistic form (Gillam and Johnston, 1992) or the phonological difficulties the DLD group faces.

Our third hypothesis was that the DLD group would present lower performance than the TD group in the total number of different words used in their texts. According to the results of our study, there was a statistically significant difference between the two groups in terms of lexical diversity, which is in line with a number of previous findings. More specifically, our results are consistent with those of Machie et al. (2013) who also presented evidence that the DLD group in their study produced fewer different words while writing than TD peers matched in age. Also, Dockrell and Connelly (2012) indicated in their study that DLD individuals used fewer different words than their TD peers and Williams et al. (2013) also evidenced a limited number of different words in the writing task of their DLD group compared to that of TD peers. Additionally, this finding is in line with Ralli et al. (2021) study in the Greek language which revealed that the DLD group produced fewer different words than their TD peers of a corresponding chronological age. The DLD group in all the above studies mentioned tended to use a significantly less diverse range of words than their TD peers of the same chronological age that probably indicates vocabulary deficits. Previous research has documented that limited vocabulary knowledge may impact on the ability to create ideas, a fact which leads to use a restricted number of words in written outputs (Williams et al., 2013).

Our fourth hypothesis was that the DLD group would present lower performance than the TD group in the total amount of nouns or verbs used in written texts. According to the results of our study, a statistically significant difference was found between the two groups for noun production but no statistically significant difference was found between the two groups for verb production in written texts, thus our hypothesis was partly confirmed. Our finding that there was no significant difference between the two groups in the proportion of verbs produced agrees with that of Williams et al. (2013) but differs from the findings of Stuart et al. (2020). However, Stuart et al. (2020) highlighted that there was a statistically significant difference in the number of verbs produced between the DLD and the chronologically age-matched group (CA) but there was a repetitive use of the same verbs in the texts of both DLD and CA groups. The differences in the findings between our study and those of the study of Stuart et al. (2020) could be attributed to the fact that in their study the DLD children that took part were older than our DLD group. This means that those children were more familiar with writing processes either through extensive practice at school or following therapist’s intervention.

Our fifth hypothesis was that the DLD group will present lower performance than the TD group in the total number of clauses (main and subordinate) and in the percentage of the subordinate clauses among the total number of clauses produced in the written texts. This hypothesis was not confirmed as there was no statistically significant difference in the total amount of clauses (main and subordinate) and in the percentage of the subordinate clauses among the total number of clauses produced in the written texts between the two groups. This finding could be attributed to the fact that sentence boundaries in the texts collected by the participants were not always detectable because of the inconsistent use or/and lack of punctuation and capitalization in the written samples of both groups which made the coding of the scripts a difficult task. This difficulty was also stated by Stuart et al. (2020) who observed the same while coding their participants’ scripts. Both groups in our study produced simple sentences containing only a main clause that is consistent with the findings of Machie et al. (2013). In their study, they observed that the DLD group as well as all the control groups of the study did not use complex sentences and they attributed it to delays in the acquisition of grammatical knowledge (Machie et al., 2013). Poor sentence construction on the part of both DLD and TD participants evidenced in our study as well as in previous research could be attributed to the participants’ young age and the subsequent low level in terms of grammar and syntax.

Our sixth hypothesis was that the DLD group will present lower performance than the TD group in the total number of content and function words used in the written texts. This last hypothesis was partly confirmed. Specifically, there was a statistically significant difference in the total number of content words used but no statistically significant difference in the total number of function words used in written texts was found. The DLD group used fewer content words (verbs, nouns, adverbs, adjectives) than their TD peers which indicates poor vocabulary knowledge on their part. Furthermore, in line with Broc et al. (2013) there was no statistically significant difference in the use of function words (articles, connectors, pronouns) between the two groups. The absence of difference could be attributed to the use of short well known and frequently used function words by the DLD group. No other studies were found including data on the use of function or content words in written text production so as to compare the findings of this study with previous ones.

In this study, we examined written language production in the Greek language of children with DLD as compared to that of their peers of the same chronological age, in terms of the words (total number, incorrectly spelt or different) produced, in the total number of nouns and verbs used, in the use of clauses (main and subordinate) and in the content and function words used. In the majority of our measures, the DLD group performed poorer than their TD peers. This finding indicates that the DLD group faces difficulties not only in oral language, which most of previous research has focused on, but in their written language production as well, in the Greek language, in which research concerning the writing performance is limited. Therefore, our findings provide useful information concerning the performance of DLD individuals in Greek, a language with rich morphology, that differs from English which most previous research has focused on. The challenge for researchers and educators is to understand the type of errors DLD children usually make while writing the Greek language and try to practice more on them.

However, our results should be treated with caution since there are some limitations in the present study that need to be considered. A limitation of the study is the small number of the participants in both the DLD and the TD groups, although a number of studies used similar or smaller samples. For example, Mackie and Dockrell (2004) included 33 children in their study (a group of 11 DLD children, a group of 11 TD children matched for age, and a group of 11 children of a corresponding linguistic age) while Ralli et al. (2021) included 60 children (a group of 30 DLD children and a group of 30 TD children of a corresponding chronological age) in their study. In general, small samples do not allow generalization of the findings which need to be replicated with larger samples in order to be confirmed. In addition, our study was limited to only one writing sample per participant which included a limited number of words in most cases and the participants were assessed only once at a specific time. Therefore, follow up studies are needed in order to replicate our findings. A further limitation of our study is that the DLD group was recruited from speech therapy centers which means that these children might have been benefited from intervention programs they followed. In addition, the fact that the DLD group did not have a language-matched or reading-matched control group, but only a chronologically matched group, consists another limitation of the study as we cannot conclude whether the written language production is similar to that of younger children with similar profile (language or reading). Also, the research with language-matched or reading-matched control groups could highlight whether difficulties with written language production could be attributed to the individual’s level of language development. In conclusion, further research is needed with larger samples, chronologically as well as language-matched or reading-matched, who will be followed longitudinally on a number of writing samples in Greek. In addition, detailed analysis of the errors made in the Greek language should be conducted which will provide useful information on the type of errors made in Greek by DLD individuals that could be used for designing intervention programs targeted specifically for this group, with the purpose of improving their written language abilities.

## Data Availability Statement

The raw data supporting the conclusions of this article will be made available by the authors, without undue reservation.

## Ethics Statement

Ethical review and approval was not required for the study on human participants in accordance with the local legislation and institutional requirements. Written informed consent to participate in this study was provided by the participants’ legal guardian/next of kin.

## Author Contributions

GA and VA conceived and designed the study, drafted, and edited the manuscript. All authors contributed to the article and approved the submitted version.

## Conflict of Interest

The authors declare that the research was conducted in the absence of any commercial or financial relationships that could be construed as a potential conflict of interest.

## Publisher’s Note

All claims expressed in this article are solely those of the authors and do not necessarily represent those of their affiliated organizations, or those of the publisher, the editors and the reviewers. Any product that may be evaluated in this article, or claim that may be made by its manufacturer, is not guaranteed or endorsed by the publisher.
